# The Mystery of Piezophiles: Understudied Microorganisms from the Deep, Dark Subsurface

**DOI:** 10.3390/microorganisms11071629

**Published:** 2023-06-22

**Authors:** Gabrielle Scheffer, Lisa M. Gieg

**Affiliations:** Department of Biological Sciences, University of Calgary, Calgary, AB T2N 1N4, Canada; gabrielle.scheffe1@ucalgary.ca

**Keywords:** piezophile, deep terrestrial subsurface, deep marine subsurface, piezophile adaptations, extremophile, high pressure, microbial biogeography

## Abstract

Microorganisms that can withstand high pressure within an environment are termed piezophiles. These organisms are considered extremophiles and inhabit the deep marine or terrestrial subsurface. Because these microorganisms are not easily accessed and require expensive sampling methods and laboratory instruments, advancements in this field have been limited compared to other extremophiles. This review summarizes the current knowledge on piezophiles, notably the cellular and physiological adaptations that such microorganisms possess to withstand and grow in high-pressure environments. Based on existing studies, organisms from both the deep marine and terrestrial subsurface show similar adaptations to high pressure, including increased motility, an increase of unsaturated bonds within the cell membrane lipids, upregulation of heat shock proteins, and differential gene-regulation systems. Notably, more adaptations have been identified within the deep marine subsurface organisms due to the relative paucity of studies performed on deep terrestrial subsurface environments. Nevertheless, similar adaptations have been found within piezophiles from both systems, and therefore the microbial biogeography concepts used to assess microbial dispersal and explore if similar organisms can be found throughout deep terrestrial environments are also briefly discussed.

## 1. Introduction

The definition of an “extreme” condition is anthropocentric, meaning that it is centered on regular human conditions, and that which deviates from these conditions is considered to be extreme. Bacteria and Archaea are microorganisms that, over millennia, have adapted to many abiotic pressures that are considered to be extreme, such as high or low temperatures (below 20 °C or over 45 °C), high pressure (over 0.1 MPa), high salinity (over 1.2%), exposure to radiation, or even exposure to toxic concentrations of metals [1]. One of the extreme conditions that has been far less studied compared to the others is the adaptation of these microorganisms to high-pressure environments, which are largely found within deep marine and terrestrial subsurface environments.

Organisms that have the extraordinary capacity to withstand high pressure were discovered more than 130 years ago, but progress in their study has suffered somewhat from the requirement of specialized techniques for the collection of samples and enriching microbial communities while maintaining a high-pressure environment [2,3]. In the late 19th century, Certes analyzed some sediments from the Travailleur and Talisman expedition and questioned the importance of microorganisms from the deep sea to transform organic matter [2,4]. He also was able to show that certain microorganisms can grow under high pressures. In 1949, the term “barophilic” first appeared to describe organisms that were pressure-adapted [5]. In 1957, Zobell and Morita developed a titanium vessel resisting high pressures of up to 100 MPa to study these pressure-loving organisms [6]. Decades later, in 1979, the term “barophilic” was re-termed “piezophilic” after the first isolation of a pressure-adapted organism by Yayanos (Table 1) [7]. Most known piezophilic organisms have been discovered within the last 40 years, making this field of environmental microbiology research relatively new [8].

The goal of this review is to summarize the current state of knowledge regarding the adaptations used by various types of microorganisms living at high pressures and to highlight the knowledge gaps in this field of research. As piezophiles often simultaneously belong to other extremophile groups (such as psychrophiles, thermophiles, or halophiles), the need for proper controls is also discussed. Furthermore, the microbial biogeography of the deep marine and terrestrial subsurface is discussed in the context of whether both habitats could encompass similar organisms or at least similar adaptations to high-pressure environments. Lastly, challenges in sampling and cultivating piezophilic microorganisms are highlighted. 

## 2. Provenance and Description of Piezophiles

Microbiological studies of piezophiles have been conducted using samples that originate from different environments, and studies contributing to knowledge related to cellular adaptations under high hydrostatic pressure have focused on both bacterial and archaeal isolates (Table 2). In this section, we briefly overview the two main habitats from which piezophiles have been isolated—the deep marine subsurface and the deep terrestrial subsurface—with various adaptations used by microorganisms recovered from these environments highlighted in Section 3. 

### 2.1. Geological Provenance of Microorganisms

#### 2.1.1. Deep Marine Environments

**Figure 1:**
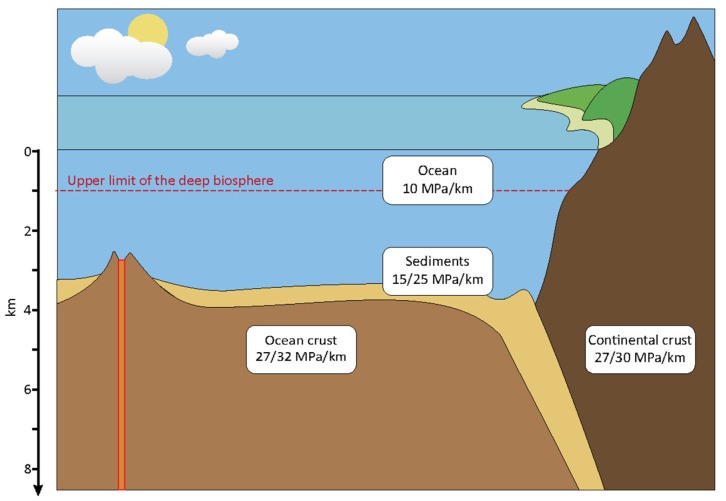
Schematic showing different deep biosphere environments and associated pressures (Modified from Oger and Jebbar [45]. Copyright © 2010 Elsevier Masson SAS. All rights reserved.).

Because 71% of the Earth’s surface is covered in water, it is no surprise that most successfully laboratory-isolated piezophilic organisms have been retrieved from the deep marine subsurface [2]. The oceanic minimal limit to which piezophiles can grow at 10 MPa or higher is found at a depth of 1000 m (Figure 1) and accounts for 88% of the volume of the ocean [8]. At even deeper depths, the pressure can range from 10 MPa up to 100 MPa at the deepest location of the ocean, the Mariana Trench (11,034 m deep) [2]. The average hydrostatic pressure of the deep sea is around 38 MPa, at an average depth of 3800 m [8]. Pressures can be higher than 100 MPa in the subseafloor, composed of marine sediments for the initial 500 m of the oceanic crust as an average [2]. Examples of deep hydrosphere environments would be the deep sea, subsurface marine sediments, and the oceanic crust (Figure 1 and Table 2).

#### 2.1.2. Deep Terrestrial Environments

The lithosphere is delimited to the oceanic crust and the upper mantle (Figure 1), which may contain fossil fuels [46]. The pressure within the continental subsurface was reported to be an average of 30 MPa·km^−1^ [47]. Examples of deep terrestrial environments include crude oil reservoirs, mines, deep terrestrial igneous rocks, aquifers, and groundwater. To date, comparatively little research has been performed on piezophiles within this environment, and most publications discussing deep terrestrial organisms are recent. Some studies have highlighted the adaptations of different microorganisms from deep oil reservoirs and Deccan Traps (deep igneous rocks; Table 2) [38,39,40]. Other subsurface studies have been performed on deep terrestrial igneous rocks (basalts and granite-gneiss rocks), African gold mines, Finnish Pyhäsalmi mines, Polish deep-subsurface hot brines, and groundwater samples (including sands and gravel aquifers), but these studies focused on microbial diversity rather than on piezophile adaptations (Table 2) [41,42,43,44,48,49].

### 2.2. Microorganisms Adapted to Pressurized Environments 

**Figure 2:**
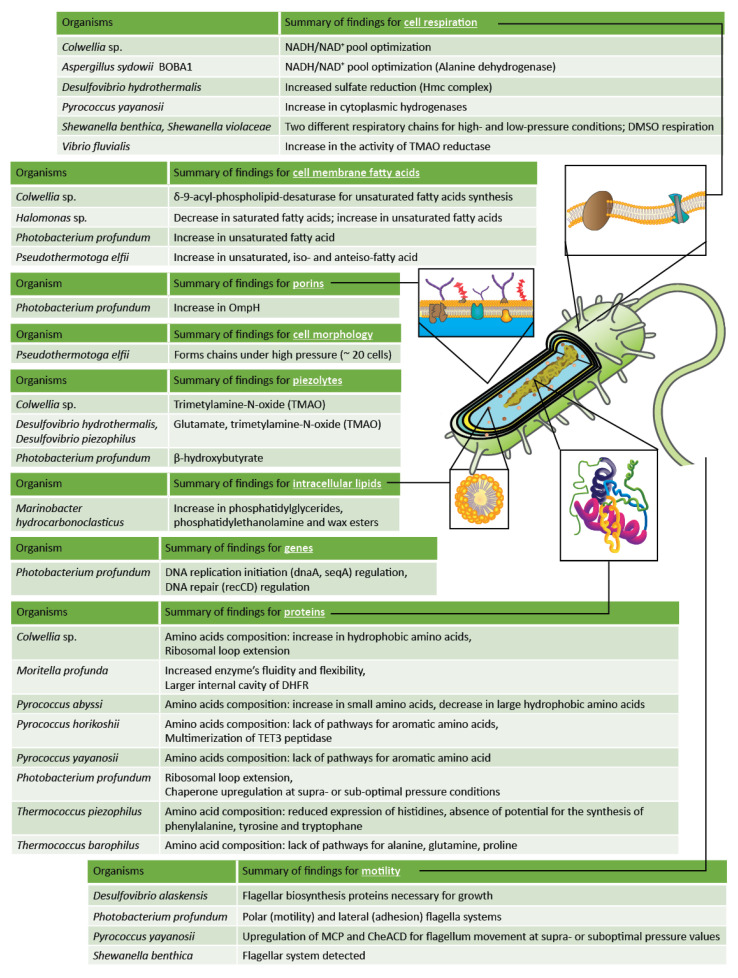
Summary schematic of adaptations in piezophilic microorganisms related to biomolecules and other microbial functions. Adaptations are reported for exposure to high-pressure conditions unless otherwise indicated. Note that the prokaryotic cell, as drawn, serves as a general model here and does not depict any specific microorganism detailed in this review.

A recent review article indicated that >80 piezophile isolates had been reported (as of March 2021) [50]. In Figure 2, we summarize isolates for which adaptations to high-pressure conditions have been reported and that are discussed in this review. Other studies have focused on microbial diversity rather than piezophile adaptations and can be consulted for more information (Table 2) [8,24,41,42,44,50,51]. Interestingly, most microorganisms adapted to life in cold to moderate temperatures are bacteria isolated from deep marine environments; almost all organisms found in high temperature environments have been found associated with deep-sea hydrothermal vents and are members of the Archaea domain [12]. As an exception, a recent study described a thermophilic bacterium retrieved from an oil reservoir (65 °C), an environment that has yet to be thoroughly investigated as a high pressure and high temperature environment [38]. Some organisms that are found in the deep sea but are not specifically adapted for deep-sea extreme conditions can be introduced by the sinking of phytoplankton debris or as a spore [51]. 

## 3. Current Knowledge of High-Pressure Cellular Adaptations in the Deep Biosphere

This section overviews the current knowledge surrounding various cellular adaptations of piezophiles, ranging from cell motility to adaptations in DNA. While we do recognize that high-pressure habitats can be characterized by different environmental conditions (such as temperature or redox conditions) that will influence these adaptations, for the purposes of this review, we organized the discussion of piezophilic adaptations based on organisms (bacterial and/or archaeal) retrieved from the deep marine environments and the deep terrestrial environments without specific taxonomic distinction (e.g., organized based on habitat rather than taxonomic distinction). The subsurface environments and specific organisms highlighted in this review are shown in Table 2. 

It should be noted that most research to date on the topic of piezophiles has been performed using microorganisms retrieved from the deep marine subsurface environments; thus, less is known about adaptations in piezophiles retrieved from the deep terrestrial subsurface. However, adaptations reported for terrestrial microorganisms are highlighted where available in the following sections. 

### 3.1. Motility, Chemotaxis, and Biofilm Adaptations

#### 3.1.1. Adaptations in Microorganisms Retrieved from the Deep Marine Subsurface 

Motility is a known phenotype of organisms in the ocean that is used for nutrient acquisition, as well as to limit predation from protozoa [30,52,53]. It was observed that the obligate piezophile *Pyrococcus yayanosii* experienced an upregulation of genes coding for chemotaxis pathway when exposed to pressure that was less than and greater than the optimal growth pressure value of 28 MPa, promoting motility [9]; specifically, the proteins MCP and CheACD were upregulated. MCP, a methyl-accepting chemotaxis protein, acts as a stimulus receptor and allows the phosphorylation cascade for the CheACDY proteins to activate of the movement of the archaellum [9,54]. Eloe et al. [33] also reported two flagellar gene clusters in the piezophile *Photobacterium profundum* SS9 that were absent in the pressure-sensitive strain 3TCK. It seems that, for this organism, one flagellar system is a polar system used for motility in liquid environments, and one is a lateral flagella system that is well-adapted to high-pressure conditions, thus helping with the translocation on surfaces or viscous media [33,55,56]. Interestingly, contradictory results were obtained from a study performed with *Nautilia* sp. PV-1 where a proteomics analysis showed a decrease in flagellin expression under high pressure [15].

#### 3.1.2. Adaptations in Microorganisms Retrieved from the Deep Terrestrial Subsurface

Motility has also been reported as an adaptation of piezophiles inhabiting the deep terrestrial environment. A study of the piezophile *Desulfovibrio alaskensis,* originally isolated from an oil-well corrosion site in California [39], found that genes involved in flagellar biosynthesis were essential for this sulfate-reducing bacterium to grow under high pressure [39]. When studying mutants in flagellar biosynthesis genes, motility and growth under high hydrostatic pressure were negatively impacted. Specifically, when the mutant strains ∆*flaB3*, ∆*fliD*, and ∆*fliA* were exposed to high hydrostatic pressure, all were non-motile and had a lower average growth rate compared to the non-mutant strain. *FlaB3*, *fliA,* and *fliD* genes encode flagellin proteins (FlaB3) and regulators of flagellar assembly (FliA and FliD) [57,58]. The authors suggested a positive relationship between high pressure, motility, and biofilm formation in this sulfate-reducing bacterium [39]. 

While motility through the expression of flagellar systems is common to numerous taxa, research to date has suggested its importance for piezophilic growth. Flagellar upregulation was reported to be higher or lower than the ideal growth pressure in *P. yayanosii*, upregulated at optimal growth pressure in *P. profundum* and *D. alaskensis*, and absent in *Nautilia* sp. strain PV-1. More research needs to be performed on these microorganisms to fully understand the circumstances under which motility is linked to high hydrostatic pressure. 

### 3.2. Cell Morphology Adaptations

#### Adaptations in Microorganisms Retrieved from the Deep Terrestrial Subsurface

The most recent research on pressure tolerance in deep crude-oil reservoirs compared *Pseudothermotoga elfii* DMS9442, a piezophile that grew optimally at 40 MPa, and *Pseudothermotoga elfii* subsp. *lettingae*, a piezo-sensitive organism that grew optimally at atmospheric pressure [38]. *P. elfii* DMS9442, when grown at 40 MPa, showed an interesting phenotype. When observing the cells under a microscope, the authors found that a variable proportion of cells formed chains from 0.07% at 0.1 MPa up to 44% at 40 MPa. In the case of the non-piezophile *P. elfii* subsp. *lettingae*, the proportion of chained cells always remained low. The number of cells per chain also increased with increased hydrostatic pressure, reaching a maximum of 15 cells at 40 MPa for *P. elfii* DMS9442, compared to ~5 cells per chain for the piezosensitive *P. elfii* strain [38]. Furthermore, more cell death occurred at a high hydrostatic pressure for cells that were not part of cell chains, and the authors reported that this was the first time that a morphotype of *Pseudothermotoga* was characterized. While the authors did not identify the biological mechanisms involved in the process, they hypothesized that the cell chain arrangement might favor intercellular communication, possibly as part of an energy-saving strategy [38]. 

### 3.3. Cell Membrane Adaptations 

When living under high hydrostatic pressure, the cell membrane loses fluidity by compacting the fatty acid chains. Membrane functionality is preserved by increasing the proportions of unsaturated fatty acids in their lipids, resulting in the disorder of the membrane [1,45]. 

#### 3.3.1. Adaptations in Microorganisms Retrieved from the Deep Marine Subsurface

An increase in the degree of unsaturated fatty acids in cell membranes was observed in a study conducted by Kaye and Baross [14], where temperature, salinity, and pressure effects were investigated on four different strains of *Halomonas*, which included non-piezophiles and piezophiles. In this study, hydrostatic pressure was reported to be the dominant condition affecting membrane fluidity. It was observed that the proportion of saturated fatty acids decreased as the pressure increased (conversely, the amount of unsaturation increased), specifically for the 18:1ω7c fatty acid and monoenoic fatty acids [14]. A similar observation was found with the psychropiezophile *Photobacterium profundum*, for an increase of ω-3 polyunsaturated fatty acids was found in response to high hydrostatic pressure [30]. This feature was controlled by the *pfa* operon, which encoded an ω-3 polyunsaturated fatty acid synthase. Unfortunately, Campanaro et al. [30] could not obtain pressure-sensitive mutants when deleting the operon from the genome, thus limiting the outcome of this discovery. Furthermore, this operon was later characterized by Peoples et al. [24] as an adaptation to cold environments, since all strains of the psychrophilic *Colwellia*, whether adapted to high pressure or not, contained the operon which they reported as necessary to introduce unsaturated bonds to the cell-membrane fatty acids. It is therefore unclear if the *pfa* operon responds to a change in high pressure or low temperature, conditions which have similar effects on organisms. On the other hand, only piezophilic *Colwellia* had genes coding for a δ-9-acyl-phospholipid-desaturase, promoting unsaturated fatty acid synthesis [24].

An interesting trait of *P. profundum* is its increase in several outer membrane proteins (OMPs) in response to increasing pressure, specifically OmpH [29,31,59]. Upon the discovery of this OMP, Bartlett et al. speculated that porin channels functioning at low pressure would be negatively impacted by the hydrostatic pressure, hence the need for high-pressure-resisting porins enabling the transport of molecules such as amino acids and sugars into the periplasmic space [29]. The *ompH* gene is under the control of the toxR regulon, a transmembrane protein known to sense environmental changes in osmolarity or pH. When hydrostatic pressure increases from 0.1 MPa to 28 MPa, the abundance of OmpH on the outer membrane has also been found to increase approximately 10-to-100-fold [29,35,60]. 

#### 3.3.2. Adaptations in Microorganisms Retrieved from the Deep Terrestrial Subsurface

Just like deep-sea piezophiles, similar membrane adaptations were found within the cell membranes of deep-oil-reservoir-derived strains studied to date. An increase in branched iso- and anteiso-fatty acids and in unsaturated long-chain fatty acids were observed at high pressure compared to atmospheric pressure conditions for two *P. elfii* strains [38]. 

### 3.4. Energetic and Respiration Adaptations

#### 3.4.1. Adaptations in Microorganisms Retrieved from the Deep Marine Subsurface

A well-studied cellular adaptation for bacteria living under high pressure is within the respiratory chains of *Shewanella violaceae* and *Shewanella benthica*, where two respiratory chains are regulated in response to pressure. In these *Shewanella* spp., one of the respiratory chains becomes significantly altered under extreme conditions [27,45,61]. At low pressure (0.1 MPa), three respiratory chain enzymes are present: NADH dehydrogenase, the bc-1 complex, and the terminal cytochrome c oxidase [61]. At high pressure (60 MPa), the last two enzymes are different from the low-pressure respiratory chain: a membrane-bound cytochrome c-551 and a terminal quinol oxidase [61]. The quinol oxidase is expressed only under high-pressure conditions, while the cytochrome c oxidase is present only under atmospheric pressure [45].

For members of the *Colwellia* genus [24], an additional NADH ubiquinone oxidoreductase gene cluster was found in these bacterial piezophiles that was absent in mutants and non-piezophiles. This is a unique NADH dehydrogenase which allows for the translocation of four protons per two electrons, which may assist the organism in energy acquisition when experiencing high hydrostatic pressure. Another finding was the identification of an alanine dehydrogenase unique to piezophiles that allows the reversible amination of pyruvate to alanine that also oxidizes NADH to NAD^+^ + H^+^. This enzyme was also found within the fungus *Aspergillus sydowii* BOBA1 grown under high-pressure conditions [36]. The authors postulated that this unique NADH dehydrogenase may help under extreme conditions to preserve the homeostasis of the NADH/NAD^+^ pool. However, this adaptation was found within organisms that are psychrophiles; therefore, it may not be a unique characteristic of piezophilic organisms alone [24]. 

Another type of respiration that seems to have adapted to bacterial piezophile organisms is dimethyl sulfoxide (DMSO)-induced reduction for its use as a terminal electron acceptor. When combining various pressures and temperatures, it was shown that low temperature or high pressure allowed for the type I DMSO system to be expressed, while a higher temperature or lower-pressure condition allowed for the type II system expression [37]. The type I system is localized at the outer membrane and matches the *dmsEFABGH* organization of *Shewanella*, while type II may be localized in the periplasmic space and is more similar to that of *E. coli* [37,62]. A study performed on isolates of the bacteria *Vibrio fluvialis* QY27 and ATCC33809 showed that when trimethylamine N-oxide (TMAO) was used for energy metabolism, the strains showed a piezophilic phenotype (growth at 30 MPa). The authors noted an increase in the activity of the TMAO reductase under high pressure [22]. 

When examining the respiratory pathways within the archaeon *Pyrococcus yayanosii*, Michoud and Jebbar observed that the energy pathway utilizing hydrogenases coupled with formate metabolism was downregulated under high pressure [9]. Formate is oxidized to CO_2_ by a formate dehydrogenase, and then protons are reduced to dihydrogen by a hydrogenase in response to formate accumulation during fermentation in *Thermococcales* [9]. Furthermore, the hydrogenases that are downregulated under high hydrostatic pressure are the membrane-bound [NiFe] hydrogenases (*mbh* operon) that convert hydrogen ions to dihydrogen [9,19,63]. Out of 13 hydrogenase genes investigated, only an NADPH-specific cytoplasmic [NiFe] hydrogenase was found to be upregulated under high hydrostatic pressure. However, the research team could not identify the role of this hydrogenase under extreme conditions since its deletion from the genome did not interfere with the strain’s ability to grow [9]. Other hydrogenases regulated by the *mbx* operon showed constitutive expression regardless of low- or high-pressure conditions. Based on these results, authors have stipulated that the *mbh* and *mbx* hydrogenases represented a form of respiration in piezophilic *P. yayanosii*, allowing for the creation of a proton gradient across the membrane by accepting electrons from a ferrodoxin [9].

#### 3.4.2. Adaptations in Microorganisms Retrieved from the Deep Terrestrial Subsurface

As for respiratory metabolism adaptations from the deep terrestrial subsurface, the energy metabolism of *Desulfovibrio hydrothermalis* under high hydrostatic pressure showed an increase in six genes that are part of the Hmc complex. This complex was studied within *Desulfovibrio* species for its implication in periplasmic hydrogen oxidation and cytoplasmic sulfate reduction [16]. 

### 3.5. Piezolytes as Adaptations

Molecules that accumulate within a microbial cell in response to high pressure were first termed “piezolytes” by Martin et al. [34]. Compatible solutes help by displacing the water molecules bound to proteins that would otherwise lead to their denaturation, a process referred to as “preferential hydration” [34,64]. 

#### Adaptations in Microorganisms Retrieved from the Deep Marine Subsurface

The phenomenon of preferential hydration was studied using *P. profundum*, for which glutamate, betaine, and β-hydroxybutyrate were detected when the organism was grown at 20 to 30 MPa (its optimal pressure range for growth) [34]. While glutamate and betaine accumulation were observed at both atmospheric and high hydrostatic pressure and, hence, cannot be confirmed to be an adaptative response to pressure, β-hydroxybutyrate accumulation was only observed at high pressure. An interesting observation made by the authors was that little-to-no β-hydroxybutyrate was produced under high hydrostatic pressure in the absence of glucose [34]. However, due to the scarcity of glucose in the deep sea, carbon sources for microorganisms in this environment commonly include petroleum compounds, proteinaceous compounds, or humic substances, not glucose [65]. Therefore, even though promising results were obtained ex situ, relying on glucose fermentation for β-hydroxybutyrate production, these findings could not explain how the organism adapted to its environment, where glucose is not a common carbon source. Other studies performed on *Desulfovibrio hydrothermalis* isolated from a deep-sea hydrothermal vent in the East Pacific Rise and on *Desulfovibrio piezophilus* isolated from the Mediterranean deep sea also showed a 2.25-fold increase in glutamate accumulation under high pressure compared to atmospheric pressure [16,23,66]. This result is contradictory to the findings using *P. profundum* wherein glutamate accumulation was not affected by increasing pressure. 

An important consideration regarding compatible solutes is that, in addition to their accumulation under high hydrostatic pressure, high salinity and high temperature conditions can also lead to their accumulation by certain microorganisms. In some cases, the effects of high salinity, high temperature, and high pressure appear to be opposite to each other [14,34]. Increasing pressure leads to a favorable reaction with regards to protein hydration, while high salinity and high temperature have the opposite effect. In the case of high-pressure conditions, the process is thought to involve water molecules which penetrate the center of a protein, resulting in its denaturation [34]. This process is referred to as the “destruction of voids”. In the case of high temperature or salinity, reduced water activity has numerous negative effects on proteins, including incorrect folding [8]. Although these effects are different, the accumulation of compatible solutes allows for adaptation to harsh environments. Different compatible solutes are unique to different environmental conditions. For example, mannosylglycerate, di-myo-1,1-inositol phosphate, and diglycerol phosphate are signature compatible solutes commonly found in thermophiles to stabilize and preserve the function of proteins experiencing high temperatures [12,67,68]. However, mannosylglycerate also accumulated in response to high salt stress in the hyperthermophile *Pyrococcus* [8,12]. Glutamate and betaine are other compatible solutes accumulated by halophiles, but they are also present when piezophiles experience high pressure and/or high-salinity conditions [8,34,69]. Trimethylamine-N-oxide (TMAO) is used for energetic purposes in some piezosensitive organisms, imparting increased tolerance to pressure [22], but the absence of reduction genes within some piezophiles indicates its use as a piezolyte at least for *Colwellia* species and *Photobacterium profundum* [24,34]. Hence, special attention and rigorous controls must be used to confirm which condition is truly causing the accumulation of a certain solute, or when studying cell membrane adaptations where inconsistencies are highlighted (see Section 3.3.1; *pfa* operon under cold or high-pressure conditions).

### 3.6. Intracellular Lipid Adaptations

#### Adaptations in Microorganisms Retrieved from the Deep Marine Subsurface

Most of the research regarding lipid composition of piezophiles has been focused on the cell membrane specifically (Section 3.3). However, it should be noted that intracellular lipids can also be modified under high pressure, as reported for *Marinobacter hydrocarbonoclasticus* [21]. Compared to atmospheric pressure, an increase in phosphatidylglycerides and phosphatidylethanolamine abundances were found when the strain was incubated at 35 MPa. Unsaturated wax esters were also more abundant, representing around 46% of the wax ester types compared to 3% at atmospheric pressure [21]. These results indicate an increase in the ratio of unsaturated intracellular lipids, similar to that found within cell membrane adaptations where an increase of unsaturated cell membrane lipids was highlighted. 

### 3.7. Protein Adaptations

#### 3.7.1. Adaptations in Amino Acid Composition

##### Adaptations in Microorganisms Retrieved from the Deep Marine Subsurface

Amino acid alterations to proteins have also been reported as a piezophile adaptation [11]. Reed et al. reported that the amino acid composition within the proteome of *Pyrococcus abyssi* showed an increase in small amino acids (serine, glycine, valine, and aspartic acid), with an equivalent reduction in amino acids with large hydrophobic residues (tryptophan and tyrosine) in the core of proteins [70]. This was also reported by Michoud and Jebbar who found the genome of the piezophile *Pyrococcus yayanosii* to lack several pathways for aromatic amino acid synthesis, such as tryptophan [9]. Similar results were found when Moalic et al. [19] studied the transcriptome of *Thermococcus piezophilus*. A decrease in the expression of histidines and the absence of potential for the synthesis of phenylalanine, tyrosine, and tryptophane was reported [19]. Reed et al. reported the amino acid adaptation as advantageous because it would allow for tight packing under high pressure conditions [70]. This is contradictory to what was reported by Ichiye, who postulated a higher stability of enzymes when larger cavities were present in enzymes [71]. Gunbin et al. designed an ideal experiment where they compared the genomes and proteomes of three different strains of *Pyrococcus* that have similar temperatures, pH values, and salinity optima but different pressure requirements for growth that correlate with their in situ habitat depth [10]. The first interesting observation was the loss of metabolism for aromatic amino acids by the piezophile *P. horikishii*, which was also observed in the other piezophilic *Pyrococcus* sp. [9,13]. Gunbin et al. [10] also examined the change in frequency of amino acids between the strains. They detected an increase in arginine, serine, glycine, valine, and aspartic acid and a decrease in tyrosine and glutamine for piezophiles *P. abyssi* and *P. horikishii* [10], as was also reported in other studies [9,11]. Cario et al. [20] further reported that another piezophile, *Thermococcus barophilus*, did not require three amino acids for growth at high pressure: alanine, glutamine, and proline. 

Work performed by Peoples et al. [24] showed that piezophilic members of the Gammaproteobacteria have more basic proteins than piezosensitive organisms. This observation included organisms from genera such as *Colwellia, Pseudomonas,* and *Shewanella* [24]. Peoples et al. also reported that piezophile proteins tend to be enriched in hydrophobic residues, including tryptophan, tyrosine, leucine, phenylalanine, histidine, and methionine, compared to their piezosensitive counterparts [24]. This is contradictory to findings gleaned from some piezophilic *Pyrococcus* species which lost the ability to synthesize tryptophan [9,11,24]. These contradictory results allude to different amino acid composition adaptations between archaeal (*Pyrococcus*) and bacterial genera (*Colwellia, Psychromonas,* and *Shewanella*). 

#### 3.7.2. Adaptations in Multimerization 

##### Adaptations in Microorganisms Retrieved from the Deep Marine Subsurface

Another way that proteins have adapted to high hydrostatic pressure is by forming multimeric proteins. One example of this is the TET3 peptidase from the piezophile *Pyrococcus horikoshii*. This protein forms a dodecamer rather than a barrel-shaped multimer, which was shown to increase stability under high pressure (50 MPa) [13,70]. The conformation of the protein makes the individual monomers more compact, making it less likely for water molecules to penetrate the core of the molecule under high pressure [70]. Once again, this is contradictory to what was reported by Ichiye [71], who reported an increase in protein stability with an increase in core cavities. Nevertheless, this conformation also appears to protect the hydrogen bonding between protein subunits.

#### 3.7.3. Adaptations in Protein Structure and Conformation 

##### Adaptations in Microorganisms Retrieved from the Deep Marine Subsurface

It was previously discussed that the cell membranes of piezophiles show increased fluidity and flexibility in response to compression from high hydrostatic pressures. A similar pattern has been shown for several enzymes. Dihydrofolate reductase (DHFR) is a necessary enzyme involved in the synthesis of purine and some amino acids, and it is therefore widespread within all microorganisms [26,71,72]. When Ohmae et al. [25] examined the structural differences of DHFR between a piezosensitive *E. coli* strain and the piezophile *Moritella profunda*, they found that the structure of the enzymes from both strains overlapped almost perfectly. When examining this result further, the authors found that two tryptophan residues were substituted by phenylalanine and valine in DHFR in *Moritella profunda* compared to that of *E. coli* [25]. The DHFR from the piezophile *Moritella profunda* was found to be less stable at atmospheric pressure than *E. coli*’s homolog DHFR protein, despite their structural similarities. Similar results were found within *Shewanella benthica*’s DHFR [71]. This lower stability (and hence high flexibility) is an advantage that has been reported for different psychropiezophiles [25,71,73]. 

In response to the increased compaction of proteins, another adaptation involves the larger cavity volume of proteins to make them more compressible and helps to prevent their distortion [71]. One example is the increased internal cavity volume of piezophilic *Moritella profunda*’s DHFR at 340 Å compared to *E. coli*’s DHFR at 270 Å [25,71]. 

Another important consideration for piezophiles is maintaining catalytic activity, which is highly impacted by temperature and pressure. For example, the catalytic activity rate of most deep-sea psychropiezophiles reported by Ichiye [71] was four-to-five times higher than that reported for *E. coli*’s DHFR. This implies that even if the activity is reduced by the high pressure and low temperature, the catalytic activity can still enable bacterial growth under these conditions [71]. The increased catalytic activity is due to a decrease in activation enthalpy [74]. 

#### 3.7.4. Ribosome Adaptations

##### Adaptations in Microorganisms Retrieved from the Deep Marine Subsurface

Another adaptation identified in some piezophiles within the Gammaproteobacteria is the extension in the loops of the 16S ribosomal molecules [45]. A study amongst members of this class (*Photobacterium, Colwellia*, and *Shewanella*) showed disparities between sister strains from different depths. These differences come from a few insertions within the loop 10 and 11 of ribosomal proteins. These insertions led to longer stems in the loops that are exclusively featured in piezophiles from this class when screening 800 members of Gammaproteobacteria [75]. This feature is consistent with observations from piezosensitive *E. coli*, where the 30S ribosomal subunit was shown to dissociate under high pressure. The hybrid construction of the piezotolerant *Pseudomonas bathycetes* 30S ribosomal subunit with the 50S subunit of *E. coli* showed greater tolerance to hydrostatic pressure [75,76]. Another study compared the ribotypes of *Photobacterium profundum* and showed that the ribotypes with longer loop stems were directly correlated to the optimal growth pressure of organisms (r^2^ = 0.97). A similar observation was also found for piezophilic *Shewanella* species [75]. 

#### 3.7.5. Chaperone Adaptations

Chaperones are proteins that are involved in the folding of proteins and help prevent the unfolding, disassembly, or aggregation of proteins under denaturing conditions [77]. Hence, chaperones have been reported to be upregulated under high hydrostatic pressure to help in maintaining protein folding in piezophiles [1,45].

##### Adaptations in Microorganisms Retrieved from the Deep Marine Subsurface

When studying the response of *Photobacterium profundum* under supraoptimal conditions, Campanaro et al. found that chaperone proteins were upregulated [30]. These proteins included *htpG, dnaK, dnaJ*, and *groEL,* which are known to be involved in preventing the protein aggregation of denatured proteins under stress; they also degrade mutated or abnormally folded proteins [30]. The opposite results were found for *Nautilia* sp. PV-1, for which *groEL* was downregulated at a high hydrostatic pressure [15].

##### Adaptations in Microorganisms Retrieved from the Deep Terrestrial Subsurface

Similar to what had been found for deep marine subsurface organisms, a bioinformatic study performed on piezophiles from water samples in the Deccan Traps also showed an increase in an abundance of genes encoding chaperones (*dnaK, dnaJ, groEL,* and *xlpPX*) [40].

### 3.8. DNA Adaptations

#### 3.8.1. Adaptations in Microorganisms Retrieved from the Deep Marine Subsurface

The effect of hydrostatic pressure of *Photobacterium profundum* on DNA replication mechanisms was also studied [32]. El-Hajj et al. studied the genes *dnaA* and *seqA* under atmospheric and high pressures to see how DNA replication was affected within this organism. DnaA is responsible for the initiation of replication by binding to the origin of replication, while seqA is a negative regulator of the initiation of DNA replication by binding to oriC and preventing the binding of DnaA [32,78,79]. A *seqA* mutant was found to be unable to grow at atmospheric pressure, but that growth was greatly enhanced at high pressure [32]. The absence of a negative regulator appeared to help with the initiation of DNA replication and therefore aided the growth of *P. profundum* under extreme conditions [32]. 

RecCD is known to be essential for double-stranded DNA repair in the case of breaks [80]. *RecD* mutants in the piezophile *Photobacterium profundum* SS9 made the strain pressure sensitive [32]. Unfortunately, beyond this observation, the damage signal for pressure-induced double-stranded DNA breakage is unknown [3]. Unfortunately, the scarcity of publications examining the regulation mechanisms for replication under high hydrostatic pressure limits the knowledge in this field. 

#### 3.8.2. Adaptations in Microorganisms Retrieved from the Deep Terrestrial Subsurface 

From the Deccan Traps’ metagenomes, genes known to be involved in DNA repair were found (*mutT, recD,* and *uvrAD*) [40]. *MutT* genes are important to avoid replication mistakes within genomes [81], while *uvrAD* is necessary for DNA repair under ultraviolet-light exposure [82]. 

### 3.9. Overall Summary of High-Pressure Adaptations in Microorganisms

Adaptations for microorganisms found in high-pressure environments in the deep marine and terrestrial subsurface have been reported, and these include modifications to features such as motility, cell membrane unsaturation, respiratory pathway adaptations, the upregulation of chaperones, and gene regulation. Due to there being more available works in the literature regarding deep marine subsurface organisms, additional adaptations have been reported for organisms from this environment, including the production of piezolytes, intracellular lipid adaptations, and a variety of protein adaptations. However, cell morphology adaptations have been reported from deep terrestrial environments only. 

## 4. Microbial Diversity in the Deep Biosphere

One of the most controversial assertions in microbial biogeography comes from Martinus Willem Beijerinck, who stated that “everything is everywhere, but, the environment selects” [83]. This statement implies that microorganisms are ubiquitous but that the environmental conditions select for their presence or absence [83]. As overviewed above, piezophiles appear to have similar adaptations (based on studies conducted to date) regardless of whether they inhabit the deep marine or deep terrestrial subsurface. Hence, one can speculate that similar microorganisms can be found in both deep marine subsurface environments and deep terrestrial subsurface environments. Key concepts of microbial community assembly that can be used to answer this question are based on Vellend’s conceptual synthesis of community ecology, relying on diversification, dispersal, selection, and drift (Table 3) [84,85]. Interestingly, dormancy (i.e., endospores) has been shown to be independent of all of these concepts. Endospores were shown to be unaffected by unfavorable abiotic conditions and even favored during microbial dispersal to which exposure to many various abiotic conditions is possible [86,87]. 

Thus, would similar or different piezophiles be expected to inhabit the deep marine and terrestrial subsurface? In this case, the concept of selection would be of no help, as the pressure and temperature gradients within the deep marine and terrestrial subsurface are reported as being similar (i.e., increasing with depth) [2], and both ecosystems are vast environments where abiotic conditions are highly variable. Both environments are expected to be inhabited by piezophiles also adapted to high temperatures or even high salinity, but can one know for sure whether similar species will be present?

One driving component of a microbial community composition that is very important for considering the answer to this question is dispersal. The topic of microbial dispersal is highly debated currently. While some authors have argued that geographical barriers are common and very important for bacterial evolution (i.e., genetic drift; Table 3), other studies have shown the transport of bacteria over thousands of kilometers, from the Atlantic Ocean, crossing the Indian Ocean, and to the Pacific Ocean [86,87,89]. Logically, environments such as surface soils, plant leaves, or streams are more likely to experience microbial dispersal (through wind, water, and/or animal transport) than the deep terrestrial subsurface or from marine sediments that are usually mostly isolated environments [87]. 

Aquifers are part of the deep terrestrial environment, to which a connection between this environment and the oceans (deep marine subsurface) was recently made [90]. This could be a potential conduit by which microorganisms could be dispersed from the terrestrial to the marine environment, although no works from the literature were found to describe this process, and only speculations are made here. To be successfully dispersed from an aquifer to the deep marine subsurface, piezophiles must be able to withstand unfavorable conditions such as lower pressure through the passive transport; hence, endospore-forming piezophiles would be more likely to be found within the two environments [91]. Furthermore, endospores have been reported to sink from the shallow depth of the ocean to the seafloor [51]. Common piezophiles between the deep terrestrial and marine subsurface are therefore a possibility; however, this is only hypothesized here. That being said, not all subsurface environments allow for the dispersal of microorganisms. For example, deep shale oil reservoirs have limited diffusion capacities due to the submicron pore spaces within the formations [92]. Whether microorganisms can be native to shale or are introduced during human operations is not the topic of this discussion; in any case, this undisturbed environment would make dispersal very limited if not functionally impossible. These isolated environments could allow for bacterial evolution through genetic drift and diversification, in which case different species would be found between the deep marine and terrestrial subsurface [85,86]. 

## 5. Methodological Challenges in Studying Piezophiles

The study of high-pressure-adapted microorganisms is relatively new. It is estimated that 12 to 20% of all bacterial and archaeal organisms inhabit the deep terrestrial subsurface, while approximately 1.8% inhabit the deep marine subsurface [93]. With only <100 piezophiles reported to date from these high-pressure environments [50], there is a clear lack of knowledge on high-pressure-loving organisms compared to other extremophiles such as thermophiles or halophiles. Unfortunately, this is due to the high costs, difficulties with proper sampling, enrichment material, and issues related to sample contamination [3,6,94]. 

The access to subsurface samples, whether terrestrial or marine, is quite challenging compared to other more accessible surface environments and therefore limits the study of deep subsurface microorganisms, including piezophiles. In general, expensive and specialized drilling technologies are required for proper sampling, whereas more commonly pre-existing, non-specialized infrastructure is used instead (South African mines and oil-reservoir infrastructure) [3,92,94]. For oceanic subsurface samples, the most common sampler used is a multi-bottle rosette sampler, which is useful for shallow sampling and limits the contamination potential of samples [6]. Few sampling instruments allow for the preservation of both pressure and temperature conditions, and this is a challenge for piezophiles, which are usually psychrophiles or thermophiles [6,71]. The recovery of deeper rock and sediments (deeper than 300 m) requires rotary drilling combined with drilling fluids (which can be ocean water in the case of oceanic subsurface environments) [94]. The use of such drilling fluids can introduce contamination within the pristine samples due to the high density of marine microorganisms, which must be taken into consideration when studying the samples [93]. 

The preservation of samples for geochemical and microbiological signatures is also challenging. Returning marine sediment samples to the surface, for example, can take a significant amount of time, during which microbial signatures can change [94]. For many research studies, samples need to be transported by boat or plane from the sampling site, thus adding to the analysis time and ultimately resulting in shifts in the microbial community. Once the samples are recovered to the surface, contamination sources need to be controlled, including the surface water used during drilling, air contamination, or additives used in the drilling fluids [6]. Laboratory experiments to study piezophiles also require specialized equipment such as high-pressure chambers, which can be challenging for many laboratories [6]. 

## 6. Conclusions

Although the field of study for piezophiles has been limited due to challenges in sampling and cultivating organisms, many cellular adaptations have been discovered in different types of piezophilic microorganisms, as highlighted in this review. These organisms live in environments in which high pressure may not be the only extreme condition; they are also frequently exposed to high salinity and high temperature, which may have additional effects on their cellular metabolism. Proper controls and more studies need to be performed to assess the effects of these combined factors on piezophiles. Furthermore, the similarities in abiotic factors between the deep marine and terrestrial subsurface and microbial dispersal potential showed that it cannot be ruled out that similar organisms, or at least similar adaptations, could be found within both ecosystems. However, additional research to address this hypothesis is needed. Despite the discoveries overviewed here, several gaps in knowledge remain, as highlighted throughout this review. The world of high-pressure-adapted microorganisms is fascinating, with many mysteries and common metabolic themes remaining to be discovered. 

## Figures and Tables

**Table 1:** Definition of microorganisms that grow within different ranges of pressure.

Term	Definition
Non-piezophile	Microorganism that has optimal growth under atmospheric pressure and cannot grow under higher pressures
Piezosensitive	Microorganism that has optimal growth under atmospheric pressure but can still grow under higher pressures
Piezophile	Microorganism that has optimal growth under high pressures

**Table 2:** Summary of piezophiles and their respective environments that are highlighted in this review.

Marine or Terrestrial Environment?	Environment	Studied High- Pressure Adaptations or Diversity?	Organisms Retrieved	Reference(s)
**Deep marine subsurface**	Hydrothermal vents (Mid-Atlantic ridge)	Adaptations	*Pyrococcus yayanosii*	Michoud and Jebbar (2016) [9]
		Adaptations	*Pyrococcus furiosus*, *Pyrococcus abyssi*, *Pyrococcus horikoshii*	Gunbin et al. [10], Di Giulio [11], Martins and Santos [12], Rosenbaum et al. [13]
	Hydrothermal vents (Location not specified)	Adaptations	*Halomonas* sp.	Kaye and Baross [14]
	Hydrothermal vents (East Pacific rise)	Adaptations	*Nautilia* sp. PV-1	Smedile et al. [15]
		Adaptations	*Desulfovibrio* *hydrothermalis*	Amrani et al. [16]
	Hydrothermal vents (Italian islands)	Adaptations	*Aquifex aeolicus*	Hervé et al. [17]
	Hydrothermal vents (Mid-Cayman Rise)	Diversity	-	Dalmasso et al. [18]
		Adaptations	*Thermococcus piezophilus*	Moalic et al. [19]
	Hydrothermal vents (Snake Pit)	Adaptations	*Thermococcus barophilus*	Cario et al. [20]
	Deep sea (Mediterranean seawater)	Adaptations	*Marinobacter* *hydrocarbonoclasticus*	Grossi et al. [21]
	Deep sea (South China Sea)	Adaptations	*Vibrio fluvialis*	Yin et al. [22]
	Deep sea (Mediterranean Sea)	Adaptations	*Desulfovibrio piezophilus*	Pradel et al. [23]
	Deep sea (Mariana Trench)	Adaptations	*Colwellia* sp.	Peoples et al. [24]
	Marine sediment (West African Coast)	Adaptations	*Moritella profunda, Moritella yayanosii*	Ohmae et al. [25], Penhallurick and Ichiye [26]
	Marine sediments (Ryukyu Trench)	Adaptations	*Shewanella benthica*, *Shewanella violaceae*	Yamada et al. [27], Zhang et al. [28]
		Adaptations	*Photobacterium* *profundum* SS9	Bartlett et al. [29], Campanaro et al. [30], Campanaro et al. [31], El-Hajj et al. [32], Eloe et al. [33], Martin et al. [34], Welch and Bartlett [35]
	Marine sediments (India)	Adaptations	*Aspergillus sydowii* BOBA1	Ganesh Kumar et al. [36]
	Marine sediments (West Pacific)	Adaptations	*Shewanella piezotolerans*	Xiong et al. [37]
**Deep terrestrial subsurface**	Oil reservoir (Location not specified)	Adaptations	*Pseudothermotoga elfii* DMS9442	Roumagnac et al. [38]
	Oil reservoir (USA)	Adaptations	*Desulfovibrio alaskensis*	Williamson et al. [39]
	Deccan Traps (India)	Adaptations	*Methylotenera* sp., *Caulobacter* sp., *Alcanivorax* sp.	Dutta et al. [40]
		Diversity	-	Dutta et al. [41]
	Hot brines (Poland)	Diversity	-	Kalwasińska et al. [42]
	Gold mine (Africa)	Diversity	-	Takai et al. [43]
	Pyhäsalmi mines (Finland)	Diversity	-	Miettinen et al. [44]

**Table 3:** Definition of concepts related to microbial community assembly [85,86,88].

Term	Definition
Dispersal	The transport of microorganisms by wind, water, or other macro-organisms
Diversification	The evolution and adaptation of microorganisms through horizontal gene transfer or mutations, for example
Drift	The changes in the relative abundance distribution of a microbial community
Selection	The effect of abiotic factors (pH, temperature, salinity, pressure, availability of carbon) selecting for the microbial community structure

## Data Availability

No new data were created or analyzed in this study.
